# Postural influence on intracranial fluid dynamics: an overview

**DOI:** 10.1186/s40101-023-00323-6

**Published:** 2023-04-13

**Authors:** Arlan Faritovich Sagirov, Timofey Vladimirovich Sergeev, Aleksandr Vladimirovich Shabrov, Andrey Yur’evich Yurov, Nadezhda Leonidovna Guseva, Elizaveta Aleksandrovna Agapova

**Affiliations:** Department of Ecological Physiology, Federal State Budgetary Scientific Institution “Institute of Experimental Medicine”, 12 Academic Pavlov St, Saint-Petersburg, 197022 Russia

**Keywords:** Postural changes, Craniospinal system, Brain blood flow, Intracranial pressure, Intracranial compliance, Cerebral autoregulation, Cerebrospinal fluid, Head-up tilt, Head-down tilt

## Abstract

This review focuses on the effects of different body positions on intracranial fluid dynamics, including cerebral arterial and venous flow, cerebrospinal fluid (CSF) hydrodynamics, and intracranial pressure (ICP). It also discusses research methods used to quantify these effects. Specifically, the implications of three types of body positions (orthostatic, supine, and antiorthostatic) on cerebral blood flow, venous outflow, and CSF circulation are explored, with a particular emphasis on cerebrovascular autoregulation during microgravity and head-down tilt (HDT), as well as posture-dependent changes in cerebral venous and CSF flow, ICP, and intracranial compliance (ICC). The review aims to provide a comprehensive analysis of intracranial fluid dynamics during different body positions, with the potential to enhance our understanding of intracranial and craniospinal physiology.

## Background

During a passive shift in body position from vertical to horizontal, various physiological processes occur, including the redistribution of the blood in vessels and cerebrospinal fluid (CSF) in subarachnoid spaces and ventricles, heart deceleration, decrease in blood pressure, adaptive reactions from the autonomic nervous system, and changes in impulse patterns from muscle proprioceptors and vestibular apparatus. Anthropologically, these changes are of particular interest in studying human adaptive capabilities in environments with different degrees of gravitational force, while medically, they provide an opportunity to learn more about pathological instances resulting from dysregulation of normal adaptive mechanisms or preexisting maladaptation. The implications of exposure to different degrees of gravity on the human organism have been a main focus in space biology and medicine. Although several studies have extensively researched intracranial hydrodynamic shifts and the development of orthostatic hypotension during prolonged space flights [1–3], human body reactions to postural changes in the context of nervous and cardiovascular diseases, such as arterial hypotension, hydrocephalus, and high intracranial pressure of various causes, are not extensively studied.

Postural changes, mostly orthostatic ones, were examined in hemodynamic and hydrodynamic aspects by direct pressure measurement inside CSF spaces of the craniospinal axis, Doppler ultrasonography, and MRI. Antiorthostatic tests or head-down tilts are limited by studies aimed at simulating environmental features, such as microgravity and weightlessness, to learn their impact on the human body. Nonetheless, the results and conclusions obtained from these trials can be applied to a variety of cases, such as the assessment of autoregulation of cardiovascular and cerebral hemodynamics and their influence on CSF circulation and brain activity, exploration of pathological conditions related to elevated intracranial pressure (ICP), and potential practical value in the complex application of ortho- and antiorthostatic tests for diagnostics and rehabilitation or adaptation to space conditions.

This review focuses on studies that describe the flow dynamics of blood and CSF in the brain during various types of body positions, with a detailed look at methodological approaches used in those studies that enhanced the quality of the results. Throughout the review, the evidence that touches upon the interrelations between CSF flow and arterial and venous blood flow, as well as their respective volumes, is discussed. In the conclusion part, a hypothesized model of their flow and volume dynamics during various postural tilts is introduced.

The objective of this review is to collect and introduce data from several scientific works dedicated to matters in intracranial and craniospinal physiology and the postural impact of different body tilts on CSF flow, cerebral arterial (CaBF) and venous blood flow (CvBF), ICP, intracranial compliance (ICC), and other parameters reflecting the intracranial environment. In the conclusion, a comprehensive picture of the intracranial fluid dynamics influenced by postural changes is attempted, elaborating more on the acute effects of short-term ortho- and antiorthostatic positions.

This review’s many suggestions can serve as an additional guide for creating novel methods that assess and analyze the relationship between CSF circulation and cerebral and cardiovascular hemodynamics, which can shed more light on human adaptability in space and other environments with different gravitational forces, as well as provide practical solutions for medical and research purposes.

### Cerebral autoregulation and hemodynamic effects of short- and long-term postural changes

The total cerebral blood flow (CaBF) through the internal carotid (ICAs) and vertebral arteries (VAs) in the supine position is reported to be around 750–870 ml/min on average [4–6]. This flow is tightly regulated by cerebral autoregulation (CA), which is a physiological process that maintains adequate oxygenated blood supply to the brain in response to changes in cerebral perfusion pressure (CPP), mainly by regulating the resistance level of the blood vessels. CA also regulates the amount of blood present in the brain, which is a major compensatory mechanism that protects the brain from high intracranial pressure (ICP) [7, 8]. Normally, an increase or decrease in CPP leads to vasoconstriction or vasodilation. CA is activated within 2–5 s after changes in CPP, stabilizing it in the range of 50–150 mmHg [8–11].

There are two approaches to evaluate CA: static and dynamic. The static approach assesses steady-state changes of CaBF relative to arterial blood pressure, while the dynamic approach represents CaBF response to rapid shifts in blood pressure and the time it takes to return to baseline. CA partly depends on the intrinsic mechanisms, including the direct response of vascular smooth muscle to blood pressure alterations and innervation from subcortical neurons or local cortical interneurons [12]. CA also depends on extrinsic perivascular innervation from sympathetic and parasympathetic parts of the autonomic nervous system [13, 14]. The latter participates in the phase regulation of vasoconstrictor and cardiomotor neurons located in the ventrolateral section of the medulla. The parasympathetic nervous system acts independently of the metabolic needs of the brain and almost always increases cortical blood flow [1]. Parasympathetic neurons are also thought to be less influenced by autotrophic reflexes characterized by slow axonal transport and humoral transmission of catecholamines and neuropeptides [15]. It is also assumed that the sympathetic and parasympathetic nervous systems play a significant role in CA during hyper- and hypotensive conditions, rendering adaptive protection [13].

During instances of body position changes from upright sitting or standing to horizontal supine, following mechanical stretching of vascular walls in the carotid arteries irritates sinocarotid pressure receptors. These receptors transmit electric impulses to sympathetic vasoconstrictor and cardiomotor neurons innervating resistive vessels. Consequently, these impulses inhibit vasoconstrictor and cardiomotor neurons leading to a decrease in peripheral resistance, heart rate slowing down, and reduced arterial blood pressure, which, in turn, causes a reduction of minute blood volume. Additionally, parasympathetic cardiomotor neurons also get excited, which diminishes minute blood volume even more [16, 17]. In the described sequence, intermediary elements such as the solitary nucleus, which propagate the stimulus further, and parallel controlling structures in the brain stem, cerebellum, hypothalamus, and limbic system, were omitted.

The effect of baroreflex modulation on CA is not fully understood. Still, it is known that baroreflex sensitivity is antagonistic to CA, judging by the described mechanism of their interaction. An inverse correlation between them and an enhanced sympathetic tone is consistent with lower baroreflex sensitivity [18]. The conclusion of most of these findings might be that CA mechanisms are more closely connected to the sympathetic nervous system. Although there is some evidence that myogenic influence on CA is more prevalent, this prevalence is only expressed in determining the lower limit of CA [14]. The sympathetic mechanism is the largest contributor within the active region of CA. Therefore, it has a significant involvement in normal adaptive responses of brain blood flow.

Although the supporting mechanisms of CA are generally reliable, there are instances of postural changes, such as high orthostatic stress, that can affect CaBF. Head-up tilts (HUT) of 60° or more reduce CaBF velocity, registered in the middle cerebral artery (MCA) by transcranial Doppler sonography (TCD), by approximately 15–20% [19–21]. Sato et al. emphasize that there is a difference in blood flow response in ICA and VA to orthostatic stress. In their study, CaBF was reduced mainly in MCA and ICA, while VA blood flow was unaffected. The authors conclude that this might indicate different dynamic CA responses evoked to maintain CPP in two major brain vascular areas during orthostatic stress. The same results, showing heterogeneity in dynamic CA between ICA and VA, were obtained in the study using lower body negative pressure (LBNP) [22]. LBNP of − 50 mmHg caused a reduction of 25% in ICA blood flow and no significant change in the CaBF of VA. In contrast to these results, another study by Kaur et al., which implemented moderate to severe LBNP tests up to − 70 Torr, did not find any type of heterogeneous response between ICA and VA [23]. Nonetheless, the majority of studies focused on orthostatic stress are alluding to the hypothesis that blood flow in the VAs vascular area decreases only during extreme orthostatic stress, most likely occurring in predisposed conditions of orthostatic intolerance or hypotension. In addition, studying a heterogeneous CaBF response in the intra- and extracranial arteries among young men during mild postural changes (+ 20° HUT and − 20° HDT), Ogoh et al. suggested that negative changes in ICP compensate shifts in hydrostatic pressure, thereby stabilizing CaBF in internal carotid and vertebral arteries. Meanwhile, the external carotid artery may provide a shunting function during HUT, since its blood flow is highly affected by gravity [24].

In cases of antiorthostatic postural changes, research indicates that CPP and CaBF remain relatively stable and within the normal range, regardless of the severity of short-term head-down tilt (HDT) [3, 24–27]. Recent MRI investigations have shown only a 5–6% reduction in total CaBF in response to short-term HDTs ranging from − 6 to − 15° [5, 6]. This robustness is achieved through the adaptive functions of CA, particularly through the regulation of cerebrovascular resistance, which differs depending on body orientation. Gelinas et al. used TCD to report a relative increase in vascular resistance of middle and posterior brain arteries during a 10-min − 90° HDT [3]. Kato et al. found no substantial differences in CaBF velocity in the MCA and transfer function gain between supine and HDT, only noting that the cerebrovascular resistance index was slightly higher in − 30° HDT compared to − 10° HDT [27].

In a complex MRI study, Zahid et al. applied − 15° HDT to examine CSF pulsatile flow, demonstrating a small 6% decrease in average blood flow rate through internal carotid and vertebral arteries among 15 healthy subjects [6]. Another sophisticated TCD study investigated acute responses from cerebral and systemic hemodynamics to transitions between HDT and HUT in ten healthy individuals, using CaBF velocity and blood pressure in the MCA and cerebrovascular resistance index (CVRi) to establish a proportional increase in these parameters to the degree of HDT [28]. This study used horizontal position as a reference to 75° HUT and short-term (15 s) HDT of − 10°, − 25°, and − 55°. The immediate transition from any degree of HDT to HUT caused a 10–20% reduction in calculated BPMCA and CVRi for a short-time period. However, CaBF velocity had the fastest recovery, returning to baseline within 5 s after the postural change. The authors claim that these results demonstrate the high efficiency of CA, at least under conditions of 1 G. They also accentuated the stability of the dynamic aspect of CA in healthy subjects, despite a short-lived drop in the parameters below the CA static range, which was assessed according to the CPP range of 50–170 mmHg. Therefore, the given studies consistently prove that CA in healthy individuals is robust against implemented postural transitions.

It is doubtful that the same outcomes can be expected when observing brain blood flow during long-term postural changes, such as head-down bed rest (HDBR), spanning several hours, days, or weeks. HDBR tests are usually conducted to simulate physiological conditions during space flights, where the hydrostatic pressure gradient of blood and CSF is absent, causing the pure volume pressure and pressure of surrounding tissues to come into the forefront, distributing between the lower limbs and the cranium. The following increase in intracellular and interstitial fluid volume and hemodynamic and autonomic adaptive-compensatory changes in the body is associated with an increase in ICP. These homeostatic shifts could lead to abnormal alterations in CA manifesting after spaceflights, resulting in orthostatic intolerance [2, 29, 30]. It is observed that only some of the returned astronauts experience this condition, and the onset of orthostatic intolerance upon returning to the planet’s surface depends on the time spent in zero gravity and is predetermined by gender as well as individual susceptibility to orthostatic stress.

Blaber et al. report controversial results of several studies conducted in the past on the usage of TCD during and after space flights and HDBR of − 6 to − 13°, concluding that the state of cerebrovascular autoregulatory mechanisms during these conditions remains elusive [31]. Many of the reviewed studies measured CaBF velocity in the MCA to identify CA changes in HDBR tests. However, Montero et al. found no alterations in CBF velocity in MCA during prolonged 180 min HDT, while CBF velocity was diminishing progressively, and CPP remained stable [25]. Similar suggestions about lack of precision in TCD measurement of CBF velocity were expressed by Lewis et al. in their article on transient hypotension during LBNP [32]. The main problem that is most likely responsible for these controversial measurements lies in the technique limitations of TCD. TCD can accurately assess cerebral blood velocity only if the cerebral vessel diameter does not change. Considering that the diameters of the measured arteries did change in the mentioned studies, the root of inconsistent results is obvious. It is recommended to aim for incorporation of several methodologies by combining or using instead of TCD duplex ultrasound and MRI which can measure blood vessel diameter and velocity, enabling more accurate evaluation of CA during postural changes.

A study that used phase contrast MRI to measure arterial and venous brain hemodynamics in nine healthy male participants during − 6°, − 12°, and − 18° HDT lasting 4.5 h found a significant decrease in arterial inflow in all HDT angles compared to supine, with a maximum total CaBF reduction of 23% in 12° HDT. However, during the transition to − 18° HDT, there was a sudden 9% uptick in arterial inflow, as well as a 26% surge in venous outflow [4]. The authors explain that one of the causes of this reversal could be the accumulation of metabolites and CO_2_ in the cerebrovascular system that prevail over the hydrostatic effects that decrease CaBF. Notably, CaBF in ICAs contributed the most to changes in overall arterial inflow. The most recent MRI study also described a 17% drop in ICA CaBF and a 6% increase in ICA resistive index after more than 24 h of HDT [33]. Interestingly, exposure to 3% CO_2_ did not have much effect on CaBF. These results are true only for ICAs and not for VAs, which further reinforces the suggestion that blood flow in VAs could be more strictly regulated than in ICAs.

Despite considerable changes in CaBF during long-term HDTs shown by the respective studies, Kermorgant et al. found that dynamic CA was still improved in healthy subjects after 21 days of − 6° HDBR [34]. Perhaps implementing steeper HDT angles over long periods could yield different results.

Overall, studies on the reactions of CA and CaBF to postural changes suggest that normal CA effectively regulates blood supply to the brain during orthostatic and anti-orthostatic body positions. Based on data from these studies, CA minimizes blood flow alterations in the brain and maintains stable CPP during anti-orthostatic tilts. This is particularly true for short exposures to HDT and somewhat applicable for HDBR. In addition, changes in CVRi and autoregulatory index during HDT and HDBR indicate high CA efficiency [28, 34].

This enhanced state of CA seems paradoxical, as baroreflex modulation in antiorthostatic positions, even more than in supine, should work against CA effects that raise cerebrovascular resistance through sympathetic neurons. However, a possible explanation is that stimulation from baroreceptors is insufficient to overcome sympathetic tone or CA influence, which could be positively modulated by small increases in ICP. Arbeile et al. have suggested that the increase in cerebrovascular resistance might be connected to an increase in ICP during the first days of HDT [35].

Impairment in CA or its predisposed feebleness could lead to the development of orthostatic intolerance, which may become apparent after spaceflights or HDT and HDBR tests. Therefore, CA could be balancing the sympathetic tone of ICAs and its cerebral arterial network according to the predominant modulation of baroreflex or ICP during orthostatic and antiorthostatic postural changes. CA remains mostly unchanged regardless of any type of postural changes, except for VAs, which supply a more metabolically active vertebrobasilar system and are likely in a persistent state of vasodilation due to constant sympathoexcitation. Studies have shown little to no changes in CaBF in VAs during HDTs [4, 22, 33], and a couple of articles on the peculiarities of CA in posterior cerebral artery (PCA) [36, 37] support this conclusion. However, Nakagawa et al. found evidence that vasodilation occurs in the PCA territory mainly due to higher metabolic state in the visual cortex when the eyes are open. Apparently, in a condition with closed eyes, CA in MCA and PCA operates on the same level. It is important to note that CA assessed in PCA might not necessarily represent CA in VAs, even though PCA is a terminal branch of basilar artery, which is a continuation of both VAs.

Therefore, we can see that CA strictly maintains the arterial part of brain blood volume within physiological boundaries, at least during short-term postural changes. Long periods of microgravity or HDT can alter CA responses leading to manifesting orthostatic intolerance in some individuals. In this condition CA is functioning fairly adequate during supine and HDTs; however, upon transition to orthostatic positions altered CA cannot readjust CaBF to maintain normal CPP revealing its developed maladaptation. Other intracranial fluid compartments, such as cerebral venous blood and CSF, that are not under CA management, exhibit more flexibility in their flow and volume when body position is changing, as described further in the article.

### Cerebral venous outflow in various body tilt positions

The primary pathway for venous outflow from the brain is through the internal jugular veins (IJVs), which collect blood from the superior sagittal and rectal sinuses via the transversal and sigmoid sinuses, and from the cavernous sinuses via the inferior petrosal sinuses. In a resting supine position, the mean cerebral venous blood flow (CvBF) is approximately 750 ml/min, with 70–90% of it flowing through the IJVs and the remainder through the vertebral veins (VVs) and vertebral venous plexus [4, 38–40].

However, when the body is upright, the distribution of venous flow changes dramatically. The lumen of the IJVs partially collapses due to negative pressure that occurs at this level as the body is verticalized, which raises the IJVs above the venous hydrostatic indifference point located slightly below the heart level. This leads to increased resistance to flow in the IJVs and the redirection of blood down the VVs and other vessels of the extrajugular system supported by the adjacent tissues of the neck [41–44]. As a result, general venous outflow decreases by 2–2.5 times, to a range of 280–480 ml/min in the standing position. The reduction in CvBF in IJVs can vary from 90% (around 70 ml/min) [45] to 60% (roughly 240–370 ml/min) [40, 43], whereas the CvBF in VVs either surges from 40 ml/min to 210 ml/min or remains mostly unchanged. Such variations may stem from differences in the sensitivity of the measuring equipment or methodological approaches in choosing between upright standing or sitting body positions.

The IJVs start to collapse at head-up tilt angles of 10–30°, and performing the Valsalva maneuver in upright standing opens the IJVs again, indicating that cerebral venous outflow is dependent on central venous pressure [43, 46–49]. Therefore, the IJVs serve as a differential pressure valve when the body transitions from supine to upright [26, 50, 51]. The collapse of these veins partially redirects CvBF through high-resistance VVs, deep cervical veins, and veins of the vertebral venous plexus, which splits cerebral venous pressure from central venous pressure. This also prevents a sudden substantial fall in ICP during the transition to an upright position, as ICP decreases only mildly due to partial CSF outflow to the spinal compartment and relatively small cerebral venous pressure. Thus, the collapse of IJVs greatly influences CSF dynamics in the craniospinal system in an upright posture.

Venous outflow in the brain is highly asymmetrical, as evidenced by computed and MRI venography observations. The transverse sinus has right-sided venous outflow in about 40% and left-sided in 18% of subjects examined [48]. Multiple studies consistently show venous outflow dominance in the right IJV [4, 40, 52]. This asymmetry is likely due to differences in the connections between cerebral venous system vessels that are common among the population. In a pilot ultrasonic study by Simka et al., IJV asymmetry was highlighted, revealing that the resistance to venous outflow was two times lower in the lower IJV during lateral decubitus body position than in both IJVs in the supine position [52]. Due to this asymmetrical structure, volume gains often occurred in the right IJV. This reduction in resistance optimizes cerebral venous outflow, reducing jugular reflux, which is a reverse blood current, and potentially connected to malfunctions in the glymphatic system and neurodegenerative diseases [53, 54].

It is possible that the asymmetrical venous pathways in the cranium create various venous outflow patterns. Doepp and Schreiber identified three patterns in their study article [39]. In the first type pattern, two thirds of CvBF goes through IJVs, the second type is characterized by jugular venous flow between two thirds and one third of CvBF, and the third type is defined by venous flow in IJVs of less than one third of the overall cerebral venous drainage. Using duplex color sonography on 50 healthy participants during supine, the researchers showed that the first type pattern was found in 72% of them, the second type was distributed among 22%, and the third type was present in only 6%. This data indicates that IJVs are the primary pathway for cerebral venous outflow in most horizontal body positions. However, the authors noted that CvBF in VVs did not differ significantly between the jugular first and non-jugular third drainage types. These findings suggest that extrajugular drainage pathways, such as deep neck veins, are more involved than expected and play a significant role in the dynamics of venous outflow during orthostatic positions.

It appears that the behavior of venous outflow in antiorthostatic positions is opposite to that in orthostasis. According to Arbeille et al. [35], jugular vein enlargement was significantly observed during prolonged − 6° HDT (which is essentially HDBR) and spaceflights. They reported that after 4–5 days of HDT, the cross-sectional area (CSA) in IJVs increased by 8%, after a week it grew by 49%, and even on the 42nd day of HDT, a 40% jugular vein enlargement was still observed. Similar results were reported in spaceflight, with IJV CSA increasing by 33–47% after 1 week to 6 months. However, such a significant increase in blood filling may lead to blood stagnation and brain edema. When the body is tilted to the Trendelenburg position (− 25° HDT), the IJVs’ lumen can increase by an average of 19% compared to the supine position [55]. Marshall-Goebel et al. [4] reported that the total jugular venous cross-sectional area gradually expanded from 0.6 ± 0.2 cm^2^ in the supine position to 1.3 ± 0.5 cm^2^ at –18° HDT. However, CvBF velocity in IJVs was found to be decreased by almost half at –12° and –18° HDT compared to the horizontal position. Recent MRI studies [5, 6] reported a 15–30% increase in IJV CSA, little gains in average jugular venous outflow, and a significant drop in IJV CvBF velocity during short-term HDT of –12 to –15°. These findings suggest blood stagnation, which can lead to an increase in cerebral venous pressure and, according to Davson’s equation, an inevitable rise in ICP [50, 56].

Based on the available studies, it can be concluded that posture has a significant impact on cerebral venous outflow. When the body is in a horizontal position, the majority of venous blood flows out of the brain through the IJVs, with a higher venous flow rate observed in the right-sided vessels due to the anatomy of the intracranial venous network. However, in lateral decubitus positions, most of the cerebral venous blood volume flows through the IJV on the side of the body on which the person is lying. The transition from supine to upright posture results in IJV collapse, which depends on the degree of orthostasis. In an upright posture, venous outflow becomes more prevalent in veins outside of the jugular system, such as the VVs, deep cervical veins, and vertebral venous plexus. This collapse of the IJV and the switch to highly resistive veins are believed to play a protective role in counteracting sharp falls in ICP by disconnecting cerebral and central venous pressure during upright posture.

Antiorthostatic HDTs can lead to even greater expansion of the IJVs, but not necessarily to a higher venous outflow rate, due to the influence of central venous pressure on CvBF and ICP. This phenomenon is particularly relevant to spaceflight, where weightlessness does not prevent the continuous transmission of central venous pressure to the brain, leading to increased ICP and related symptoms. Therefore, dynamic shifts in CvBF and central venous pressure during postural changes can cause interruptions or reinforcements in the hydrostatic pressure gradient, ultimately affecting intracranial pressure. In the following sections, we will discuss how CaBF and CvBF are interrelated with ICP.

### Cerebrospinal fluid dynamics, intracranial pressure, and intracranial compliance during postural changes

Originating more than two centuries ago, the Monro-Kelly hypothesis establishes clear fundamentals about the relationships between brain matter, cerebral blood volume, and CSF [57]. Alexander Monro and George Kelly in the eithteenth century established the basis of this hypothesis, which explains the behavior of liquids inside a closed space, specifically inside the cranium. Their successors later modified the hypothesis. It claims that the total volume of brain matter, CSF, and blood inside a hermetically enclosed skull is constant. None of the components can be compressed, and an increase in one of them is only possible if the other one or two are displaced outside. It is worth adding that the hypothesis has undergone a number of corrections and additions to become what it is today. In the beginning, the founders were unaware of the third fluid, liquor, or CSF. Thus, only two components were taken into account. In the next century, CSF became part of the equation of intracranial hydrodynamics through the efforts of French physiologist Magendie and English physician Burrow. Today, the Monro-Kelly hypothesis provides theoretical explanations for a variety of pathological conditions associated with high ICP, liquor leakage, brain tumors, etc.

Currently, the cranial cavity is considered a closed space with rigid walls filled normally by 85 − 87% with brain matter, 9 − 10% with CSF, and 4 − 5% with the blood [58, 59]. Overall, intracranial CSF pressure reflects ICP and is defined by the dynamic balance between CSF secretion, absorption, and flow resistance. Normal values of CSF pressure, measured in the supine position, vary between 7 and 15 mmHg in adults. CSF pressure and ICP are dependent on many factors such as systolic pulse wave, respiratory cycles, intraabdominal pressure, arousal, physical activity, central venous pressure, and body position [60, 61].

Both active body movements and passive postural changes entail a shift in the direction of gravitational force that affects the craniospinal system, which includes the brain, spinal cord, CSF, cranial and spinal subarachnoid space, and the vasculature filled with blood. This shift results in brain and spinal cord oscillations, CSF redistribution in their subarachnoid spaces, the adaptive response from cerebroarterial circulation, and alterations in cerebral venous pressure that ultimately cause changes in ICP.

**Fig. 1:**
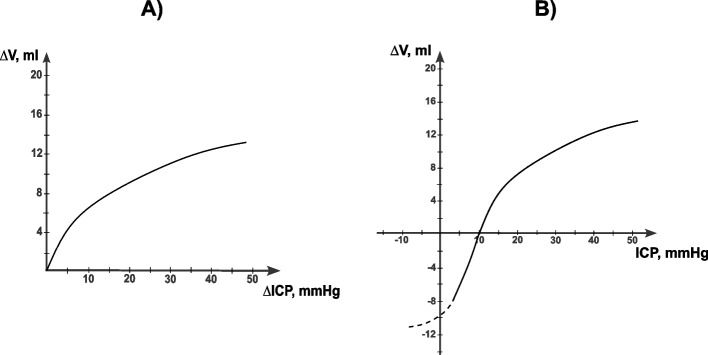
Two graph variants of the volume-pressure curve that depict ICC. **A** The classical volume-pressure curve formed by the ratio of changes in intracranial volume (∆V) to changes in ICP (∆ICP). **B** The graph with absolute ICP values on the ordinate axis instead of differential ICP values. In this graph, the volume-pressure curve and its possible continuation in a dashed line are roughly shaped according to the first graph and the sources [58, 59, 61, 62]

An important and commonly used parameter of intracranial dynamics is cerebral or intracranial compliance (ICC). Regarding the whole craniospinal system, it could also be appropriated as craniospinal compliance. ICC represents the slope of the volume-pressure curve (∆V/∆P), where changes in the intracranial volume bring reciprocal changes in ICP (Fig. 1).

**Fig. 2:**
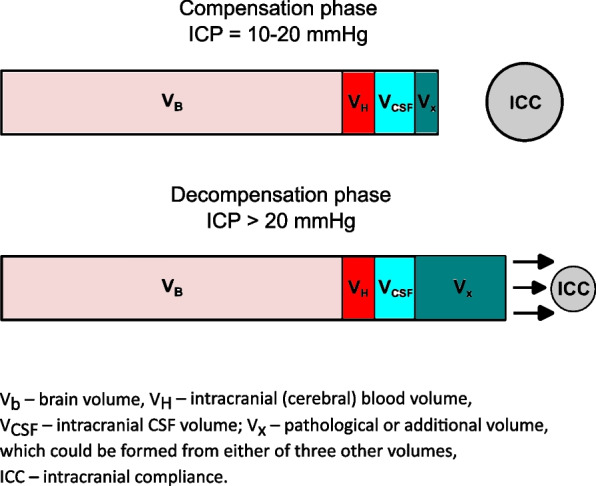
Proportional changes in volumes of intracranial compartments and ICC in spatial compensation and decompensation phases. Under normal physiological and compensatory conditions, ICC is high, indicating that there is spare space in the cranium and ICP remains relatively stable. However, in the decompensation phase, due to abnormal increases in overall intracranial volume, ICC becomes critically low, resulting in high ICP values. In this phase, any additional volume added will cause ICP to increase exponentially

The volume-pressure curve starts with the maximum values of ICC, which represents the period of spatial compensation. As more volume is infused into the subarachnoid spaces, the curve gradually rises until there is no more reserved space left, which marks the end of the period of spatial compensation. At this point, the curve sharply transitions to the spatial decompensation phase, and the ICC value rapidly becomes low [62]. The significance of ICC lies in its ability to assess the redistribution of volume in intracranial compartments, particularly in liquid compartments such as CSF, arterial, and venous blood. The pressure redistribution in these compartments follows the volume reallocation and determines the ICP at large. Moreover, ICC is primarily used as an index to demonstrate the brain’s capability to minimize changes in ICP by adjusting the volumes of intracranial compartments, mainly CSF and cerebral blood. ICC is therefore characterized as a measure of intracranial physiological reserve (Fig. 2). Average normal supine ICC values range from 1 to 1.3 ml/mmHg. ICC levels lower than 0.5 ml/mmHg are considered critical and indicate a sharp decline in intracranial reserve capabilities, regardless of baseline ICP [58, 59, 61].

It should be noted that intracranial and spinal cavities contribute unequally to the total compliance of the craniospinal system. In a study on cats, the intracranial part accounted for 68% of the total compliance in CSF spaces, while the spinal part accounted for 32% [63]. A similar proportion was found in research conducted among human subjects, with up to 50% of compensatory capabilities falling on supratentorial space, 30% on subtentorial space, and 20% in the spinal canal [58].

The first observations of changes in ICP when posture is altered were made by puncturing subarachnoid spaces to measure CSF pressure at different sites of the craniospinal axis. In 1935, Loman et al. [64] demonstrated with lumbar and occipital punctures on psychiatric patients that hydrostatic pressure decreased in the suboccipital cistern and increased in the lumbar subarachnoid space simultaneously during the transition from a supine to standing position. Similar work was done by Magnæs [65], who chose the upper point of measurement to be on the level of the lateral ventricles. On 72 control patients without intracranial pathology, he produced the same results and found that, in most cases, the zero CSF pressure point was located between the levels of the occipital protuberance and the seventh cervical vertebra. He also discovered that the hydrostatic indifferent point in CSF space, where pressure remains the same regardless of body position, stretched from C-6 to T-5 among patients in the control group.

Williams, by applying concurrent ICP measurements from ventricles and lumbar subarachnoid space in patients with various neurological conditions, established that CSF pressure is equal to atmospheric pressure in the foramen magnum [66]. Studies in neurology and neurosurgery comparing ICP values between groups of hydrocephalic patients with pressure shunts or without them and patients with normal ICP showed that the absence of a shunt predisposed to stable positive ICP measured inside ventricles regardless of postural changes. Meanwhile, shunting leads to a significant drop in ICP up to negative values in an upright position. For instance, to prove the existence of negative ICP, Fox et al. recorded ICP measurements of + 20 to + 180 mmH_2_O (+ 14.7 to + 132.4 mmHg) in the horizontal position and -140 to + 70 mmH_2_O (− 102.98 to + 51.5 mmHg) in an orthostatic position [67]. Chapman et al. reported that when patients with various shunt systems transitioned to orthostatic positions of 30 to 90^°^, their ICP inside ventricles fell to a negative range of − 15 to − 30 cm H_2_O (− 11.03 to − 22.07 mmHg) [68]. The use of an antisiphon device mitigated such a dramatic decrease in ICP, which started to vary between − 7 and + 3 cm H_2_O (− 5.15 to + 2.21 mmHg). Patients without hydrocephalus and shunts exhibited a lowering of intraventricular pressure to − 5 to + 5 cm H_2_O (− 3.68 to + 3.68 mmHg) immediately after HUT. Contemporary sources confirm that the mean negative normal ICP in an upright standing position is − 5 mmHg [26, 61, 69]. However, excluding factors such as IJV collapsibility during orthostatic position or the use of shunting systems in hydrocephalus cases, ICP could even dip to − 15 mmHg [51].

The rate of reduction in ICP depends on the degree of orthostatic tilt. Small angles ranging between 0° and 30–45° result in quick decreases in ICP proportional to the tilt, whereas high-degree angles above 45° reduce ICP slowly [50, 70, 71]. The jugular collapse, which becomes more pronounced above 30° angles, and increased intraabdominal pressure and excessive CSF filling of the spinal buffer are believed to be responsible for this disparity. This explanation could also provide insight into the differences in ICP between upright sitting and standing.

A decrease in ICP during orthostatic posture is likely to bring an increase in ICC. Several studies have investigated intracranial physiology under the impact of postural changes, and specific attention has been drawn to this prediction. Alperin et al. discovered that after transitioning from supine to upright sitting, ICC increased by a factor of 2.8 with a corresponding decrease in ICP [72]. The authors explained that these changes in parameters indicate that ICP is relatively low in the upright position because lesser blood and CSF volumes remain in respective compartments after they shifted into lower parts of the body.

Gehlen et al. introduced a generic lumped-parameter biomechanical model of the CSF and cardiovascular system [51] that used arterial blood inflow to explain the hydrodynamic physiology in supine and upright positions. The model assumed a lower spinal compliance contribution (35%) in a supine position, which further reduced in an upright posture (10%). This suggests that some portion of intracranial content is unloaded into the spinal compartment when acquiring an upright posture, leading to an increase in ICC while spinal compliance diminishes.

However, some studies have reported conflicting results. For example, a study on 13 neurological patients by Raube et al., which applied invasive intraventricular sensors, found no substantial differences in ICC during various head-up tilt tests [73]. Similarly, a more recent cross-sectional study using ICP monitoring and pulse amplitude measurements in 101 patients with suspected CSF dynamics disorders discovered that pulse amplitude was higher in sitting and standing positions compared to supine, indicating that an upright posture is associated with a minor reduction in ICC [74]. These controversial findings could be due to the fact that some studies suggesting an increase in ICC during upright are non-invasive and conducted on healthy individuals or make predictions based on a model, while other research articles report a small drop or no changes in ICC during orthostasis and use invasive measurement methods on patients with various neurological impairments.

In cases where the body is positioned antiorthostatically, a higher ICP is expected compared to supine or orthostatic positions. Studies have shown that during short-term and long-term HDT of − 6°, pressure transducers initially recorded a slightly higher ICP (Δ1.8 ± 0.5 mmHg) compared to supine, which then lowered after a few hours to match baseline supine values [75]. Non-invasive assessments of ICP alterations in − 10° HDT, through the use of arterial blood pressure and cerebral blood flow velocity, have shown that ICP could range roughly between 8 and 30 mmHg [76]. Petersen et al. demonstrated a rise in ICP in neurosurgical patients without a pathological ICP profile from 9.4 ± 3.8 mmHg in horizontal supine to 14.3 ± 4.7 mmHg in − 10° HDT and 19 ± 4.7 mmHg in − 20° HDT [26]. Another non-invasive ICP (nICP) analysis of data from 17 healthy subjects estimated 12% and 37% increases in nICP, from 7.8 ± 2.8 mmHg during a horizontal position to 8.9 ± 2.6 mmHg in − 10° HDT and 12.4 ± 3.4 mmHg in − 30° HDT [27]. Overall, antiorthostatic tilts do raise ICP, but only within normal physiological confines when considering the results of the described studies that used small angles or acute exposure to more severe tilts.

Kato et al. suggested that to prevent ICP from exceeding an upper normal limit during HDTs, CA must not be impaired [77]. This suggestion is primarily based on a systematic review that revealed a moderate to strong correlation between high ICP (≥ 20 mmHg) and impaired CA. The possibility of malfunctioning CA being the main cause of intracranial hypertension is still up for debate. Nonetheless, the severity and duration of antiorthostatic positions could certainly be contributing factors to CA destabilization and pathological increase in ICP. Case reports of robotic laparoscopic surgeries provide examples of pronounced HDTs of 30–45° that lead to abnormally high ICP values, up to the development of brain edema and various neurological complications [78, 79].

As mentioned earlier, changes in ICP are connected to corresponding changes in central venous pressure, especially during antiorthostatic tilts. Several studies have emphasized the predominant dependence of ICP during supine and HDT on central venous pressure, which impacts ICP through the venous sinuses and internal jugular veins [4 − 6, 26, 27, 44, 50, 75]. Therefore, ICP increase reflects to a large extent the rise in central venous pressure when the body is tilted downwards.

When discussing ICC, it is logical to consider its decrease during antiorthostasis as it decreases during the transition from upright to supine. If the hydrostatic pressure gradient of the venous blood and CSF column is directed towards the head, additional volume inside the cranium emerges. When compensatory reserves are filled up along with hindered CSF and venous outflow, it causes an unavoidable rise in ICP, as demonstrated by studies that used various HDTs. However, according to Lawley et al., which used ICP pulse amplitude as an indicator of ICC, it was unaltered or even slightly improved in prolonged − 6° HDT [75]. Shedding some light on this matter are MRI studies that measure CSF flow pulsations during cardiac cycles. We already discussed some of them, mainly in the section about CA. The main principle of this technique is based on the characteristics of ICP waves, which are largely modified by pulsations of intracranial arteries, and thus, ICP distribution can be estimated over the phases of the cardiac cycle [80–82].

Alperin et al. proposed the parameter of intracranial volume change (ICVC), which is characterized as the difference between the inflow and outflow of fluids into and out of the skull during the cardiac cycle. This parameter was then used to assess changes in ICP and ICC among healthy individuals, patients with intracranial hypertension, and baboons [83]. The researchers estimated an average range of ICVC in supine healthy volunteers with normal ICP to be 0.34–1.3 ml, which constituted 0.1% of the overall intracranial volume. In their follow-up study, Alperin et al. discovered that after transitioning from a supine to an upright sitting position, ICVC increased nearly twofold, corresponding to an increase in ICC [72].

Ishida et al. used a similar technique and found no significant difference in ICVC between supine and HDT of − 6° and − 12°, only a small surge in peak-to-peak pressure gradient [5]. The SPACECOT study suggested aqueductal CSF velocity amplitude for the measurement of CSF pulsatility and representation of changes in craniospinal compliance [33]. Six male participants in the study showed a 13% increase in CSF velocity amplitude in prolonged − 12° HDT compared to supine, though not statistically significant. However, brief exposure to 3% CO_2_ during HDT raised CSF velocity amplitude by 21%, which was statistically significant. Although this study did not demonstrate the definitive effect of HDT on craniospinal compliance, these results imply that if CA were to lose control and allow excessive arterial vasodilation in the brain, it would increase ICP and reduce compliance.

Mild HDTs may not be radical enough to lower ICC substantially, but even − 30° HDT only raises ICP within the physiological range unless the cerebrovascular system is altered or impaired. ICP changes between about 10 mmHg and 20 mmHg may normally only trigger minuscule perturbations in ICC, considering that ICC alters exponentially in accordance with the volume-pressure curve (Fig. 1). In the end, this assumption seems reasonable.

## Conclusion

To summarize the review, we will briefly discuss the main topics covered. The craniospinal system’s most tightly regulated part is CaBF, which CA keeps within physiological boundaries to ensure adequate CPP and normal brain function, even during postural changes. Several studies have demonstrated CA’s effectiveness during tilt-tests, zero gravity environments, and LBNP. However, impaired CA or high baroreflex sensitivity can cause noticeable shifts in CaBF, especially during high orthostatic stress. CA insufficiency has also been suggested as one of the causes of high ICP during HDTs and spaceflights. ICA and VA show heterogeneity in CaBF, with ICA mostly accounting for CaBF alterations during postural transitions due to the direct effects of baroreflex that impacts CA mitigating sympathetic tone. Unlike ICA, VA has seemingly unaffected vascular sympathetic tone and does not exhibit any large CaBF fluctuations during postural changes, LBNP, and probably microgravity.

The cerebral venous system generally reacts passively to postural changes due to bidirectional flow provided by the fixated vascular walls of venous sinuses and venous plexus of the neck. However, collapse of IJVs in orthostatic positions causes a substantial fall in CvBF that begins to flow predominantly in the extrajugular venous network. In antiorthostatic positions, CvBF increases compared to supine and orthostasis, but the rate of venous outflow remains the same or slows down depending on the degree of HDT. These changes lead to a higher cerebral venous pressure and subsequent rise in ICP.

ICP is a cornerstone of intracranial relationships between the brain, cerebral blood, and CSF. The Monro-Kelly hypothesis explains these interrelations, claiming that the total volume of brain matter, CSF, and blood inside the enclosed cranium is constant, and an increase in one of them is only possible if the other or two are displaced elsewhere. Examining ICP changes in different body positions, the ICP dependency on flow dynamics of arterial and venous blood and CSF should be considered. CvBF is directly related to cerebral venous pressure, which is the main contributor to ICP. CA, as previously stated, preserves CaBF within a certain range, modulating ICP, particularly during HDT and microgravity when it is crucial to control blood filling in the cerebral arteries and prevent elevated ICP. CSF, similarly to the venous blood in the brain, is a passive participant in the craniospinal system during postural changes. CSF influences ICP, ICC, and other intracranial physiology parameters, but to a lesser extent than CvBF. In the orthostatic body position, CSF volume mainly resides in spaces of the spinal compartment due to the direction of the gravitational vector. This results in relatively high CSF pressure in lumbar subarachnoid spaces, zero liquor pressure on the level of cervical vertebrae, and negative ICP. In supine and antiorthostatic tilts, the opposite occurs, with CSF and venous blood flowing more into the skull, leading to higher ICP and a reduction in ICC.

**Fig. 3:**
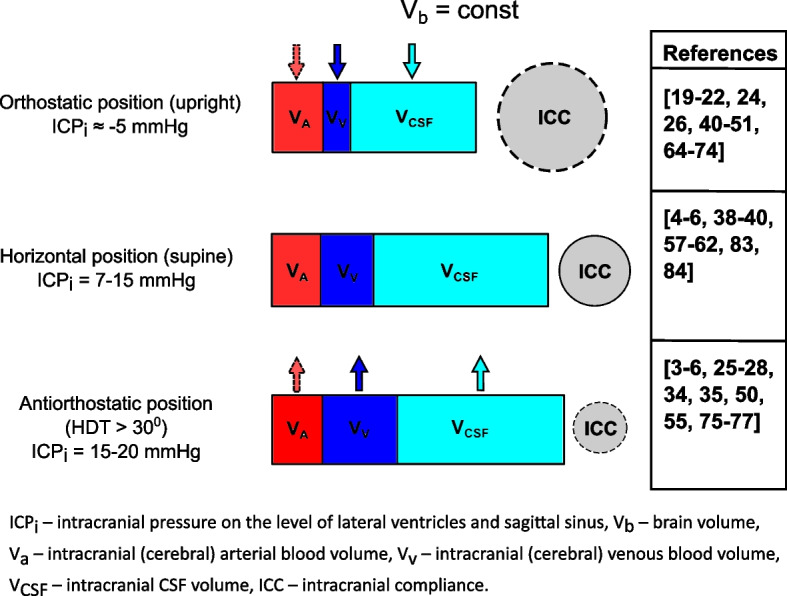
Concept diagram of potential volume interrelationships of intracranial compartments and compliance. Since brain volume does not significantly change under normal physiological conditions, it is considered constant and is not included in the diagram. The up and down arrows over the compartments indicate an increase or decrease in the respective fluid flow. The dashed arrows and lines represent hypothetical changes due to limited evidence or controversial data. On the right side, the literature studies supporting the depicted volume distribution and ICC changes are presented. The ICP values in the antiorthostatic position are assumed to increase to an upper normal limit of 20 mmHg in healthy individuals

Based on the findings and conclusions of the discussed articles and assuming normal neurological conditions, with no significant alterations in brain mass or intracranial volume, the ratios of CaBF, CvBF, and CSF flow during postural changes can provide insight into the distribution of intracranial fluid compartments (arterial and venous blood, CSF) in orthostatic, supine, and antiorthostatic body positions (Fig. 3).

Supine position is considered a reference point since normal values of ICP, ICC, CaBF, and other parameters are largely obtained in this body position, where arterial blood inflow and venous outflow should be roughly equal.

When a human body shifts from a supine to an upright standing position, there can be a progressive drop in CvBF, cerebral venous volume, and pressure due to the collapse of IJVs and passive flow of venous blood through the remaining open resistive vessels of VVs and vertebral venous plexus. Meanwhile, CaBF, depending on baroreflex sensitivity and calibration by CA, will stay within the same range as before or may reduce mainly due to decreased blood flow in ICAs. Therefore, in normal conditions, arterial blood volume will likely remain unchanged, but the predisposition to orthostatic stress may decrease it to some degree.

Intracranial CSF volume is expected to decrease as a portion of it would move into the spinal canal and spinal subarachnoid spaces. This is supported by the findings of the mentioned MRI studies on CSF flow pulsatility that demonstrated diminished oscillatory CSF volume in upright positions [72] and greater CSF volume displacement in supine positions [84]. Negative ICP values and increased ICC in the upright position also prove that some limited loss of intracranial fluid volume does occur. Additionally, due to the aforementioned events, CSF oscillation waves tend to have smaller amplitude and are less synchronized with an arterial pulse.

During antiorthostasis, changes in intracranial dynamics are opposite to those described in orthostatic positions, with some caveats. ICP is typically higher in HDTs than in supine and HUT, but it usually remains under 20 mmHg in an unimpaired craniospinal system. The ICP increment is largely connected to the rise of cerebral and central venous pressure because of the retrograde partial inflow of venous blood during antiorthostatic tilting. IJV CSA substantial expansion, slowdown of CvBF velocity, and no significant changes in cerebral venous outflow suggest venous flow stagnation in the cerebral veins. CSF exhibits similar behavior as cerebral venous blood and moves in a caudocranial direction during HDT, contributing to the general increase in ICP, though to a lesser extent. Meanwhile, CaBF is mostly stable and changes in a small range under short periods of HDTs of various degrees. CA maintains this CaBF stability through the increase of cerebrovascular resistance and by restricting the cerebral pool of arterial blood. Furthermore, the role of arterial pulsations as the driver of venous and CSF pulse waves should be even more pronounced due to an intracranial environment with higher pressure. These mechanisms play an important part in preventing pathologically high ICP and the development of neurological conditions, such as brain edema, when the body is tilted backward or during microgravity. However, there is no clear consensus among researchers on the effects of long-term HDTs, and the possibility that longer duration and steeper slope of HDT could negatively impact CA and lead to intracranial hypertension should be kept in mind. The data on antiorthostatic changes in ICC is inconsistent and suggests either that compliance is unaffected or that there are small, insignificant reductions in compliance.

In summary, by examining the effects of different body positions on CaBF, CvBF, CSF, and ICP, we can gain a comprehensive understanding of how posture impacts cerebral hemodynamics and ICP. However, it is important to note that there may be other factors that contribute to intracranial dynamics in different body positions that were not considered in this discussion. For example, the effect of central venous pressure on ICP during HDTs may be mild in rare cases of IJV valves, resulting in smaller increases in ICP. Additionally, the impact of postural changes on the rate of CSF production and absorption has not been explored. Further complex evaluation of intracranial physiology through various means under the influence of short- or long-term postural changes can result in a more detailed description of its processes and a better prediction of its changes.

## Data Availability

Not applicable.

## Abbreviations

HDT: Head-down tilt
HUT: Head-up tilt
HDBR: Head-down bed rest
LBNP: Lower body negative pressure
MRI: Magnetic resonance imaging
TCD: Transcranial Doppler sonography
CSF: Cerebrospinal fluid
ICP: Intracranial pressure
nICP: Non-invasively estimated intracranial pressure value
ICC: Intracranial compliance
ICVC: Intracranial volume change
CaBF: Cerebral arterial blood flow
CvBF: Cerebral venous blood flow
ICA: Internal carotid artery
VA: Vertebral artery
MCA: Middle cerebral artery
IJV: Internal jugular vein
VV: Vertebral vein
CA: Cerebral autoregulation
CPP: Cerebral perfusion pressure
BP_MCA_: Blood pressure in middle cerebral artery
CVR_i_: Cerebrovascular resistance index
CSA: Cross-sectional area
